# Evidence of Local Adaptation in a Freshwater Diatom Indicates Higher Sensitivity to Nutrient Limitation as Water Temperature Rises

**DOI:** 10.1002/ece3.72427

**Published:** 2025-11-09

**Authors:** Li Zhao, Divina Ryf, Sarah Levasseur, Raphaël Bossart, Marta Reyes, Frank Pennekamp, Jukka Jokela, Anita Narwani, Vanessa Weber de Melo

**Affiliations:** ^1^ Department of Aquatic Ecology Eawag Dübendorf Switzerland; ^2^ Department of Evolutionary Biology and Environmental Studies University of Zurich Zurich Switzerland; ^3^ Institute of Integrative Biology D‐USYS, ETH Zurich Zurich Switzerland

**Keywords:** activation energy, climate change, local adaptation, minimum resource requirements, phosphorus, thermal performance curves

## Abstract

Climate change is reshaping freshwater ecosystems by warming waters and modifying nutrient dynamics. These combined environmental changes exert novel gradients of selection on phytoplankton populations and communities. Temperature and phosphorus availability are individually critical determinants of growth in phytoplankton and can have interactive impacts on population and community dynamics. Although we understand how interspecific variation in thermal and resource‐use traits of phytoplankton can affect community composition in response to changing environments, the extent of local adaptation in these responses among strains of the same species remains poorly understood. In this study, we examined the local adaptation in the temperature‐ and phosphorus‐dependent growth in the freshwater diatom *Fragilaria crotonensis* . We predict that the growth of this diatom is locally adapted to the environmental conditions of the lakes of origin. To test this, we isolated strains from eight Swiss lakes (one strain per lake) with distinct historical temperatures and nutrient status. We estimated the growth rate of each strain under combined gradients of temperature and phosphorus availability. We fitted Monod curves to the growth rate data, and we quantified the minimal phosphorus requirements (P*), half‐saturation constants (*K*_s_) and maximum growth rates (*μ*_max_) for each strain as a function of temperature. We also fitted thermal performance curves and quantified activation energies (*E*_a_) and cumulative performance across the thermal gradient as a function of phosphorus availability. We observed large variation among strains in the dependence of P* on experimental temperature, with strains from phosphorus‐rich lakes showing stronger increases in phosphorus requirements with warming. These patterns imply local adaptation to phosphorus availability. Our findings highlight a potentially critical role for local adaptation in shaping phytoplankton responses to global change and call for a greater recognition of this trait variation in making predictions of community‐level responses to future climate.

## Graphical abstract

Local adaptation in thermal and nutrient‐use traits plays a critical role in phytoplankton responses to environmental change. This study investigated the local adaptation and variation in the temperature‐ and phosphorus‐dependent growth in eight strains of the freshwater diatom *Fragilaria crotonensis* from Swiss lakes, revealing that strains from phosphorus‐rich lakes showed stronger increases in phosphorus requirements with warming. These results suggest local adaptation to phosphorus availability and highlight the importance of trait diversity in predicting phytoplankton responses to combined warming and nutrient limitation.

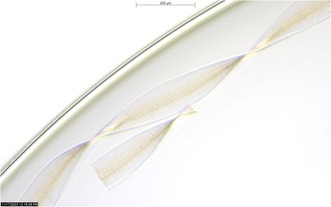

## 1 Introduction

Phytoplankton are vital primary producers of lakes and oceans (Likens 1975; Field et al. 1998) and play an important role in the biogeochemical cycles of carbon and nutrients such as nitrogen and phosphorus (Browning and Moore 2023; Hutchins and Tagliabue 2024). Climate change alters multiple environmental drivers in all aquatic ecosystems, including temperature, water quality, evaporation and resource availability (Woolway et al. 2020). Understanding how phytoplankton respond to this complex environmental change remains an urgent challenge in ecology and evolution.

Warming intensifies and lengthens periods of thermal stratification of large open water columns like deep lakes and oceans (Woolway et al. 2021; Li et al. 2020), reducing the transportation of nutrients to the surface (Yankova et al. 2017). Since essential nutrients like phosphorus and nitrogen are critical for phytoplankton growth, warming may lead to growth rate reductions because of resource limitation as waters warm. By contrast, rising temperatures alone are expected to accelerate metabolic rates (Brown et al. 2004) and thus increase nutrient demand. The temperature sensitivity of metabolism (Weber de Melo et al. 2025) and resource requirements (Levasseur et al. 2025; Lewington‐Pearce et al. 2019) can alter the identity of the best‐performing species, leading to shifts in species composition. Species better adapted to warmer, nutrient‐poor conditions may become more dominant, altering the structure and function of aquatic ecosystems (Yankova et al. 2017; Posch et al. 2012). For example, a recent study found that warm‐adapted species may have very low requirements for resources (Levasseur et al. 2025). Moreover, the “Metabolic Meltdown Hypothesis” proposes that when resource limitation co‐occurs with warming, the negative effects of elevated temperatures on ectotherm growth are amplified, because limited resources not only reduce growth rates directly but also shift the thermal performance curve (TPC), lowering both the optimal temperature for growth and the upper thermal limits (Huey and Kingsolver 2019). This suggests that under conditions of warming and low resource availability, phytoplankton growth may decline because of the combined effects of reduced energy uptake and higher metabolic costs. However, we know little about the degree to which phytoplankton strains or populations show patterns consistent with variation among species, nor whether they show signs of local adaptation to the environmental conditions of the lakes in which they have evolved.

Previous work has demonstrated that phytoplankton can evolve rapidly in response to changing temperature and nutrient availability. For example, under high temperature, the specific rates of respiration and photosynthesis of phytoplankton populations evolved in ~100 generations, leading to a 1.4‐fold increase in growth rate under 33°C, compared with the baseline of 20°C (Padfield et al. 2016). As for nutrients, similarly, the minimum phosphorus requirement of phytoplankton populations declined under phosphorus limitation, evolving in ~285 generations and adapting rapidly to nutrient‐limiting environments (Bernhardt et al. 2020). However, nitrogen‐limited conditions prevented a marine diatom from adapting to high temperature (Aranguren‐Gassis et al. 2019). These findings suggest that thermal performances and resource requirements of phytoplankton populations can evolve in response to temperature and nutrients, but the extent of intraspecific variation in responses to combined warming and resource limitation remains unexplored. Furthermore, whether intraspecific trait variation is consistent with a pattern of local adaptation to environmental conditions remains unknown. Nevertheless, one study observed variation in thermal niche width and growth rates among 12 strains of a phytoplankton species (Krinos et al. 2025), which helped this globally distributed species survive temperature changes. To better understand local adaptation and predict future distribution patterns under climate change, it is important to study the intraspecific variation in growth responses to concurrent changes in temperature and nutrient availability.

In this study, we investigate the extent of intraspecific variation in growth rates in response to combined temperature and phosphorus availability gradients. We also investigate whether this variation is associated with the local thermal and nutrient environment from which the strains were isolated, to see if these traits show signs of local adaptation. We study a common freshwater diatom, *Fragilaria crotonensis* , which is widely distributed in lake habitats across Europe, the Americas, Asia and Africa (Morales et al. 2013). *Fragilaria* species are generally known to be able to tolerate broad temperature gradients (ranging from 5°C to 30°C) (Hartig and Wallen 1986; Butterwick et al. 2004), with relatively small individual cell sizes and rapid growth rates, which makes them common contributors to spring bloom biomass, early in temperate lake seasonal succession (Lotter et al. 2010).

Different approaches can be used to identify patterns of adaptation to local environmental conditions. Here, we isolate strains from diverse habitats and test whether their best performance in laboratory experiments corresponds to the environmental conditions of their origin. This approach has successfully been employed in a previous study on *Daphnia magna* , in which 185 populations isolated from habitats varying in salinity showed salt tolerance that strongly correlated with native habitat salinity, consistent with a pattern of local adaptation in saline tolerance (Santos et al. 2024). We aimed to answer the following questions: (1) Do thermal traits depend on phosphorus availability, and do resource‐use traits depend on temperature? (2) How much intraspecific variation exists in these traits and their environmental dependencies? (3) How are these traits and their environmental dependencies related to the environment in the lakes of origin? Specifically, we hypothesized that low‐phosphorus concentrations would lead to reductions of thermal optima (*T*_opt_, the optimal temperature of growth), in line with the “metabolic meltdown hypothesis” (Huey and Kingsolver 2019) and the finding of a previous study (Bestion et al. 2018), where phosphorus limitation reduced the *T*_opt_ of several phytoplankton species. On the basis of our findings in a previous study (Weber de Melo et al. 2025), we expected that the activation energy of population growth rate (*E*_a_, which characterizes the temperature sensitivities of growth rate (Brown et al. 2004)) would be rather insensitive to phosphorus availability. We also expected a U‐shaped relationship between the minimal resource requirements of phosphorus (P*, Tilman et al. 1982) and temperature, where P* is minimized at moderate temperatures within a strain's thermal niche, and rises as temperatures deviate from this moderate value, as previously suggested (Tilman 2004; Thomas et al. 2017). Finally, we predicted that strains coming from warmer lakes would have higher *T*_opt_ values, and strains from phosphorus‐rich lakes would have higher P*s.

**FIGURE 1:**
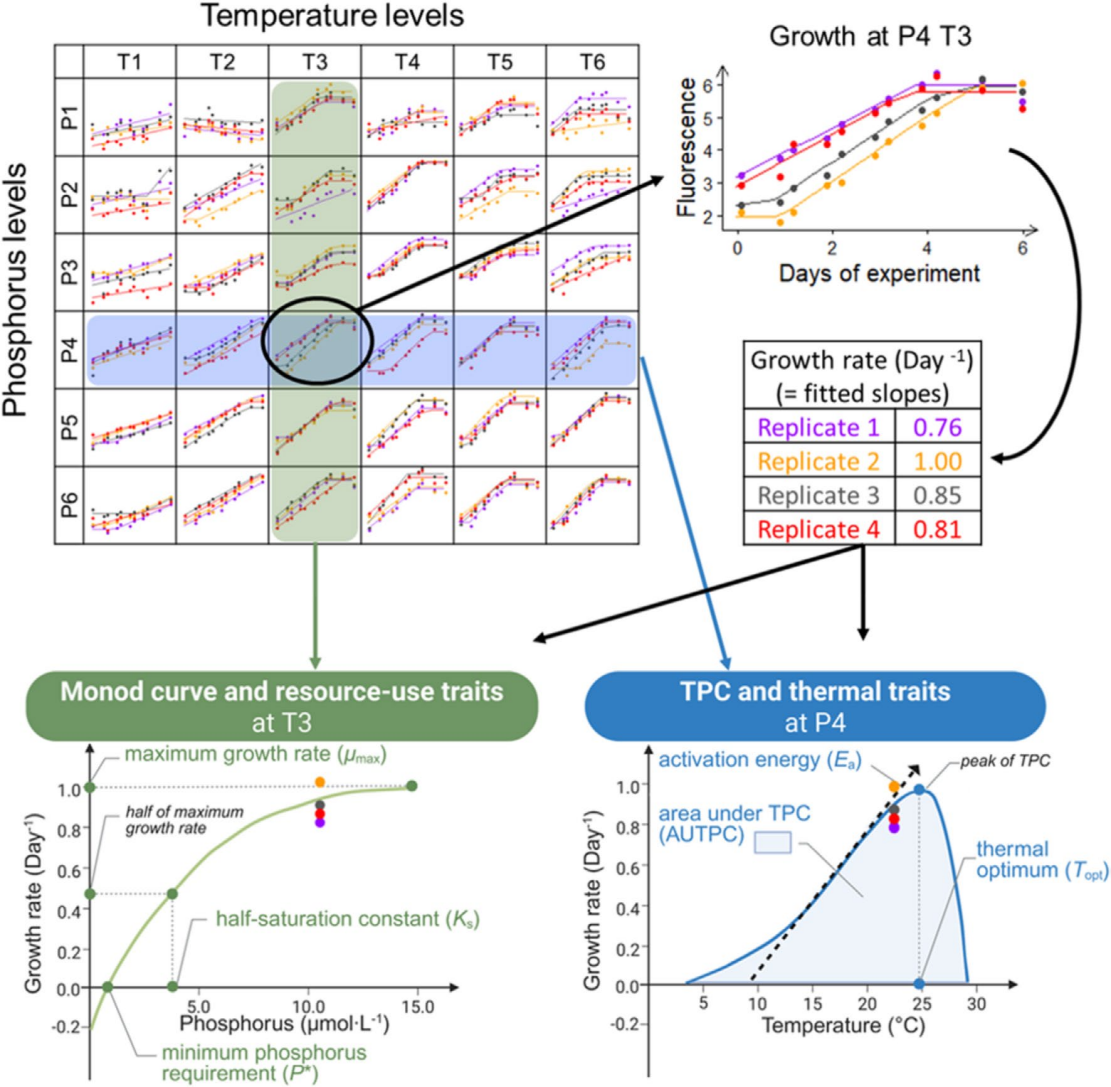
Summary of the experimental design and data analyses of the study. This conceptual figure illustrates the controlled experiment and data analysis for one strain. The same procedure was performed for each strain.

To address these questions, we performed growth rate experiments with *F. crotonensis* strains collected from eight Swiss lakes with varying environmental conditions, including temperature and phosphorus availability, to investigate how their growth rates respond to these factors. To assess thermal adaptation, we characterized each strain's TPC, which describes growth rate as a function of temperature. We also characterized the dependence of each strain's growth rate as a function of phosphorus availability using the Monod equation (Figure 1). We then investigated various descriptors of the TPCs and Monod curves that depended on the alternative gradient: for example, we investigated how thermal parameters (i.e., *T*_opt_ and the activation energy of growth, *E*_a_) depended on phosphorus availability, whereas we investigated how the Monod parameters [i.e., maximum growth rate (*μ*_max_), half‐saturation constant (*K*_s_), and minimum phosphorus requirement (P*)] depended on temperature (Figure 1). We also investigated whether there were associations of this intraspecific variation among strains with the temperature and nutrient availability in their lakes of origin.

## 2 Methods

### 2.1 Sampling and Culturing

We isolated strains of *F. crotonensis* from lake water samples collected from September to October of 2023 from 8 lakes across eastern Switzerland (Table 1), with one strain per lake. We made this choice with the aim of capturing variations in traits that result from selection under different environments, rather than within‐lake variability. Although this setup limits the insight into within‐lake diversity, it provides a controlled framework for testing hypotheses about local adaptation in response to varying lake environments. The lake samples were taken with plankton nets with a 10‐μm or 30‐μm mesh size, and then filtered with a 300‐μm mesh within a few days after sampling to remove zooplankton. After filtration, lake samples were supplemented with COMBO medium (Kilham et al. 1998) with a three‐fold concentration of silicate (300 μmol·L^−1^) and kept at 20°C. We performed serial dilutions in 96‐well plates and attempted to aspirate single *F. crotonensis* colonies from the mixture of species under a microscope, aiming to increase their relative abundance in our cultures. Single colonies of *F. crotonensis* were then isolated at the Flow Cytometry Core Facility of ETH Zurich with a BD FACSAriaIII—BSL1 sorter, using a 100 μm nozzle and standard pressure setup. The isolated colonies were grown in batch cultures in COMBO medium in 50 mL culture flasks with filter screw caps, which allow sterile gas exchange. The strains were maintained at a constant temperature of 20°C under controlled environmental conditions, with a regulated 12:12 h light–dark cycle 20 μmol photons m^−2^·s^−1^ irradiance.

**TABLE 1:** Summary of sampling location, dates and method used for collecting lake phytoplankton samples.

Lake	Sampling location	Sampling date	Sampling method
Aegeri	47°08′13.1″ N 8°35′21.1″ E	2023/10/1	Surface water with plastic bottle
Constance	47°39′00.4″ N 9°13′02.1″ E	2023/10/26	10 μm phytoplankton net, 8 m
Lucerne	Eawag Kastanienbaum	2023/10/3	10 μm phytoplankton net, 8 m
Maggiore	45°48′37.1″ N 8°36′28.2″ E	2023/10/1	10 μm phytoplankton net, surface water
Seealp	47°16′05.9″ N 9°24′01.6″ E	2023/9/24	10 μm phytoplankton net, 8 m
Walen	In front of “Camping Gäsi”	2023/9/24	7 μm phytoplankton net
Zurich	Limnological Station Kilchberg	2023/9/28	10 μm phytoplankton net, 8 m
Zug	Near Zug City	2023/10/1	10 μm phytoplankton net, 8 m

### 2.2 Species Identification and Genetic Variability

We used morphological features to identify the strains to the species level and confirm the strains as *F. crotonensis* . We prepared samples using the burn‐mount technique (Spaulding and Edlund 2021), which removes all organic material and leaves the silica frustules intact. To prepare the samples, algal strains suspended in their growth medium were placed as droplets onto microscope coverslips and heated to 500°C under a fume hood to remove organic matter (Johnson et al. 1988). Naphrax (Northern Biological Supplies Ltd., Ipswich, UK) was boiled on microscope slides and pushed against the coverslip with frustules to mount them into permanent slides, which were then observed under an inverted microscope (Leica DMi8, at 100× magnification). Morphological features of their silicon frustules, such as having 15–18 transapical stripes per 10 μm, and having a lanceolate gap between the center (2–3 μm) and the end (Huber‐Pestalozzi 1942), were used to taxonomically confirm that all eight strains were *F. crotonensis* (Figure S1).

To support the morphological identification of the strains as *F. crotonensis* , the 18S rRNA gene regions of all strains were sequenced. Additionally, genetic dissimilarities among the strains were analyzed using the 18S rRNA, rbcL, and ITS gene regions. To prepare samples for sequencing, we centrifuged a culture of each strain at 24,000 RCF for 30 min, removed the supernatant, and froze the pelleted cells. We then extracted DNA using the XS buffer method (Tillett and Neilan 2000). We amplified rbcL and partial 18S markers using multiple primers (Table S1) and then performed Sanger sequencing. Specific primers were designed on the basis of the ITS1‐5.8S‐ITS2 (Table S2), and multiple strains of each lake were sequenced using Microsynth's long PCRSeq (Oxford Nanopore Technology, serviced by Microsynth AG). These data provided an ITS consensus sequence with mapped internal mismatches, deletions, and insertions within one isolate. To lower the number of reactions needed, we created three multiplexed PCRs.

For species classification, we matched 18S amplicon sequences to database sequences for *F. crotonensis* in an NCBI BLAST search. The strains from lakes Constance, Maggiore, Zurich, Lucerne, and Zug were consistently identified as *F. crotonensis* in the top match. However, in samples from lakes Seealp, Aegeri and Walen, the two genetic markers did not consistently resolve species‐level identifications, potentially because of limitations in the 18S rRNA region's resolution for distinguishing closely related species (Table S3).

Subsequently, we investigated the genetic variability among the eight isolated strains. The 18S and rbcL regions were aligned using the Clustal Omega algorithm, and a distance tree was generated for the ITS region (Figure S2). For the rbcL region, all sequences were identical except for the strain from Lake Lucerne, which had 2.8% mismatches from the other strains. This aligns with the distance tree, showing the strain from Lake Lucerne separated from the other clusters. The ITS data revealed that the isolates from each lake were distinguishable from the others, except for the lakes Aegeri and Walen, which did not present any difference in this marker. We also sequenced multiple unique isolates per lake, and although a different number of isolates were tested for each lake (between 3 and 13 per lake), we did not observe any genetic variation among isolates within a lake (results of two representative isolates from each lake are shown in Figure S3). Strains from Lake Walen and Lake Aegeri did not show significant sequence differences in any of the tested regions. On the basis of these results, we consider that we had at least seven unique genotypes among the eight isolates from the different lakes. The two remaining strains (from lakes Aegeri and Walen) differed morphologically in cell size, with cells from Lake Walen being longer (Figure S1A,F), although they could not be distinguished by sequencing.

### 2.3 Growth Experiment

Growth experiments were performed in parallel for all eight strains in mid‐December 2023, around 2.5 months after sampling. We grew the strains under combined gradients of temperature and phosphorus concentration (Table 2), resulting in 36 unique treatments. At each treatment level, we had four replicates for each strain, which were unique experimental units grown in separate wells of the well plate. Phosphorus levels ranged between 1.94 μmol·L^−1^ and 47.47 μmol·L^−1^, whereas temperatures ranged between 12°C and 26°C. We selected these phosphorus and temperature levels on the basis of the results of a previous experiment, which was performed with the same species from Lake Constance (Levasseur et al. 2025). Our goal was to choose conditions in which growth was affected by phosphorus availability, while minimizing both levels with no growth as well as levels where growth was already saturated. Prior to the growth experiments, we gradually acclimated the strains to a light intensity of 110 μmol photons m^−2^·s^−1^, experimental temperatures (Table S4) and low phosphorus availability. Three days before the experiment, we started the phosphorus acclimation by transferring 7.5 mL of each strain into 50 mL centrifuge tubes and centrifuging at 2000 RCF for 3 min. This process was carried out in six parallel groups for each strain, corresponding to the six phosphorus levels in the experiment. We acclimated the strains to reduced phosphorus after centrifugation by removing the supernatant and resuspending the cultures in 7 mL of phosphorus level 1 media for those assigned to phosphorus levels 1, 2 and 3 in the experiment, and in 7 mL of phosphorus level 4 media for those assigned to phosphorus levels 4, 5 and 6. The resuspended cultures were transferred to culture flasks. We performed all experiments in incubators (Multitron and Multitron pro, Infors HT, Switzerland) with a 50‐rpm rotation, 12:12 h light–dark cycle and a light intensity of 110 μmol photons m^−2^·s^−1^.

**TABLE 2:** Temperature and phosphorus concentrations in the growth experiment.

Temperature levels	Temperature	Phosphorus levels	PO_4_‐P concentration	
1	12°C	1	1.94 μmol·L^−1^	60 μg·L^−1^
2	16°C	2	3.87 μmol·L^−1^	120 μg·L^−1^
3	20°C	3	7.75 μmol·L^−1^	240 μg·L^−1^
4	22°C	4	15.50 μmol·L^−1^	480 μg·L^−1^
5	24°C	5	31.00 μmol·L^−1^	960 μg·L^−1^
6	26°C	6	47.47 μmol·L^−1^	1470 μg·L^−1^

We measured the chlorophyll‐a fluorescence (excitation wavelength: 445 nm, emission: 685 nm, same for below) of the acclimated strains as a proxy for cell density before the start of the experiment. We then diluted all strains into populations with very low cell density (10 relative fluorescence units—RFU) with the media of each phosphorus level of the growth experiment. We used Freedom EVO100 TECAN automated liquid handler to transfer the diluted populations into 96‐well plates, where four replicates of each population were distributed randomly in the 32 wells in the middle of each 96‐well plate, with each replicate consisting of 200 μL. We filled the surrounding wells of the plates with Nanopure water to reduce evaporation, and all plates were covered with a sealing membrane (Breathe‐Easy, Diversified Biotech, USA). After measuring the initial chlorophyll‐a fluorescence of all populations, we monitored chlorophyll‐a fluorescence twice a day in the following 4 days, and once a day on days 5 and 6, reaching a total of 11 measurements. We measured fluorescence with a Biotek Cytation 5 plate reader.

### 2.4 Growth Rates

We omitted all fluorescence measurements lower than 2.25 RFU from our analysis since this was below the detection limit (half of the median of RFU measurements of all media with six different concentrations of phosphate). We calculated growth rates using the package “growthTools.” version 0.1.2 (ctkremer 2020). This package fits four different types of models to time series of growth and chooses the best‐fit model for each time series by their AICc scores. The models differ only in whether or not a lag and/or saturation phase is included. However, the exponential growth rate is calculated in the same way for all models, and only this estimate was further analyzed.

Across all temperatures and phosphorus concentrations in our experiment, most strains of *F. crotonensis* were able to grow, although in very few cases, strains displayed negative growth at low phosphorus levels, and at very low and high temperatures (Figure S4).

### 2.5 TPC Modeling, Area‐Under‐the‐Curve Calculation and Activation Energy Estimation

We characterized how growth rates vary with temperature, using the following formula to model the TPC:

(1) $$R={\mathrm{ae}}^{\mathrm{bT}}\left[1-{\left(\frac{T-T_{\mathrm{ref}}}{w/2}\right)}^{2}\right]$$

where *R* is the growth rate, *T* is temperature, *w* is thermal niche width (the range of temperature within which organisms can grow) and *T*_ref_ is species‐related and determines the maximum of the quadratic part of the formula (Thomas et al. 2012), with *a* and *b* as scaling parameters. We fitted TPCs with the rTPC package (Padfield et al. 2021) using the Thomas model 2012 (Formula (1), Thomas et al. 2012). We fitted multiple TPC models to our data and retained Thomas 2012 because it produced low AIC scores and showed limited risk of overfitting. We performed bootstrapping to generate 1000 randomly selected datasets, from which the upper and lower limits of the 95% quantiles of the results were taken as the confidence interval for each TPC model.

For each TPC, we calculated the area under the curve within the range of temperature in the experiments according to the trapezoidal rule (Tallarida and Murray 1987). To estimate activation energy, we applied a natural logarithm (ln) transformation of all positive growth rates in the rising part of the TPCs and re‐scaled the temperature as 1/*kT*, where *k* is Boltzmann's constant (8.62 × 10^−5^ eV K^−1^) and *T* is the temperature in degrees Kelvin. We then estimated the activation energy as the slope of the linear regression of the ln‐growth rates on 1/*kT*:

(2) $$R={\mathrm{Ae}}^{-E_{a}/\mathrm{kT}}$$

where *R* is the growth rate, *A* is a pre‐exponential factor, *E*_a_ is the activation energy, *k* is the Boltzmann's constant and *T* is the absolute temperature (Laidler 1984). We then calculated the average and the standard deviation of each strain's activation energies across all experimental phosphorus levels. For each strain, we compared their activation energies with the expectation of 0.32 eV K^−1^, which was calculated from the temperature sensitivity of Rubisco in C3 photosynthetic organisms (Allen et al. 2005).

### 2.6 Monod Curve Modeling and P* Calculation

We fitted the Monod equation to estimate growth rates along the gradient of phosphorus according to the following formula (Monod 1949):

(3) $$R=\mu_{\max}\frac{\left[S\right]}{K_{s}+\left[S\right]}$$

where *R* is the growth rate, *μ*_max_ is the estimated maximum growth rate, *K*_s_ is the half‐saturation constant, that is, the resource availability at which half the maximum growth rate is achieved and [*S*] is the concentration of the limiting resource, in our case, experimental phosphate. Minimal phosphorus requirements (P*), representing the minimum concentration of a limiting resource that a population requires to maintain a stable equilibrium population (Tilman et al. 1982), can be subsequently calculated using the estimated *K*_s_ and *μ*_max_ values, according to the equation:

(4) $$P^{*}=\frac{K_{s}\cdot \;m}{\mu_{\max}-m}$$

with *m* representing the mortality rate. The mortality rate represents an extrinsic death or loss rate, and in a chemostat model, this would represent the dilution rate, but in a more natural setting, this could represent losses to sinking, advection or other density‐independent processes. We set the mortality rate (*m*) to 0.1. Strains with lower P* can thrive better in low phosphorus environments (Tilman 1982). We fitted one model for each strain at each temperature, making a total of 48 curves. We fitted the curves using the nls.multstart package (Padfield et al. 2021) with multiple starting points to ensure robust model fitting. Boundary values were manually selected to allow sufficient exploration within biologically reasonable limits. We tested various combinations, compared their AICc values, and chose the final boundaries on the basis of the lowest AICc scores while ensuring that they did not constrain the estimation of *μ*_max_ and *K*_s_. We performed bootstrapping to generate 1000 randomly selected datasets, from which the upper and lower limits of the 95% quantiles of the results were taken as the confidence interval for each Monod curve model. To compare the original estimates with the bootstrapped values, we calculated the mean P* from the bootstrapped data and found them close to the original estimates. The strain from Lake Walen had negative P* and *K*_s_ at 12°C and 24°C, because the growth rate did not vary much along our experimental phosphorus gradient, leading to an unexpected shape of Monod curves. The *K*_s_ and P* values were excluded from further analyses because negative values are not biologically meaningful.

### 2.7 Generalized Additive Models of Thermal and Resource‐Use Traits

We used generalized additive models (GAMs) to describe each strain's response of resource‐use traits to temperature and also to describe the response of the thermal traits to phosphorus. We fitted GAMs with the R package mgcv (Wood 2017), using a Gaussian distribution and a smooth factor of *k* = 3. We used restricted maximum likelihood for smoothness selection to minimize the effect of local maxima and minima. We used the estimated degrees of freedom (EDF) of the fitted GAMs to describe the relationships between the traits and the abiotic factors; EDF values close to 1 indicate linear relationships, and larger values (e.g., ≥ 2) indicate non‐linear relationships (Hunsicker et al. 2016; Zuur 2009).

### 2.8 Correlation of Resource‐Use and Thermal Traits to Lake Environmental Data

We obtained the lake environmental data from the water temperature and phosphorus concentration records of the lakes, which were regularly monitored by cantonal environmental offices, research institutes and other organizations in Switzerland (Table S5). We used measurements taken at 5 m depth, as this is relevant for phytoplankton growth and data were available from this depth for almost all lakes, except Lake Maggiore, where only lake data of integrated surface water (0–25 m) were available.

For each lake, we calculated the mean of all temperature measurements in August, September and October (corresponding to the growing season of lake diatoms and our sampling period) for each year from 2018 to 2022, and obtained the average across years as the lake temperature. For the lakes with low monitoring frequency (lakes Aegeri, Lucerne and Walen, Table S5), we used multiple linear regression models to predict the lake temperature at a finer temporal resolution. Those models consist of a linear relationship between temperature and year, and sinusoidal terms representing seasonal variations of temperature. We then calculated the mean for each year from six predicted data points evenly distributed between August and October. We also calculated lake phosphorus concentration using the same method and time range. However, we did not compensate for low monitoring frequency with modeling, as contrary to temperature, phosphorus concentration did not fluctuate across the sampling season. Out of eight lakes in our experiment, we were unable to obtain the water chemistry data of Lake Seealp; thus, this strain was excluded from these analyses. The calculated temperature and phosphorus had a positive correlation (*p* < 0.05).

We fitted linear regression models to assess the relationship between the strains' traits and lake environmental data. We extracted both the fitted slopes and their standard errors from each regression model. We obtained the fitted slopes and significance, plotted the slopes against the experimental temperature or phosphorus levels, and fitted weighted linear regressions between them, where weights were calculated as the inverse of the squared standard errors (1/SE^2^) of the slopes from the initial regressions. This weighting approach gives greater influence to slope estimates with higher precision (smaller standard errors) and reduces the influence of less precise estimates, thereby improving the reliability of our inferences about how environmental factors modulate trait relationships. This is to investigate, for example, whether the influence of lake phosphorus concentration over resource‐use traits changes with experimental temperature. We performed all data analysis using the R programming language, version 4.3.2 (R Core Team 2024).

## 3 Results

### 3.1 Phosphorus Sensitivity of Thermal Performance Curves

**FIGURE 2:**
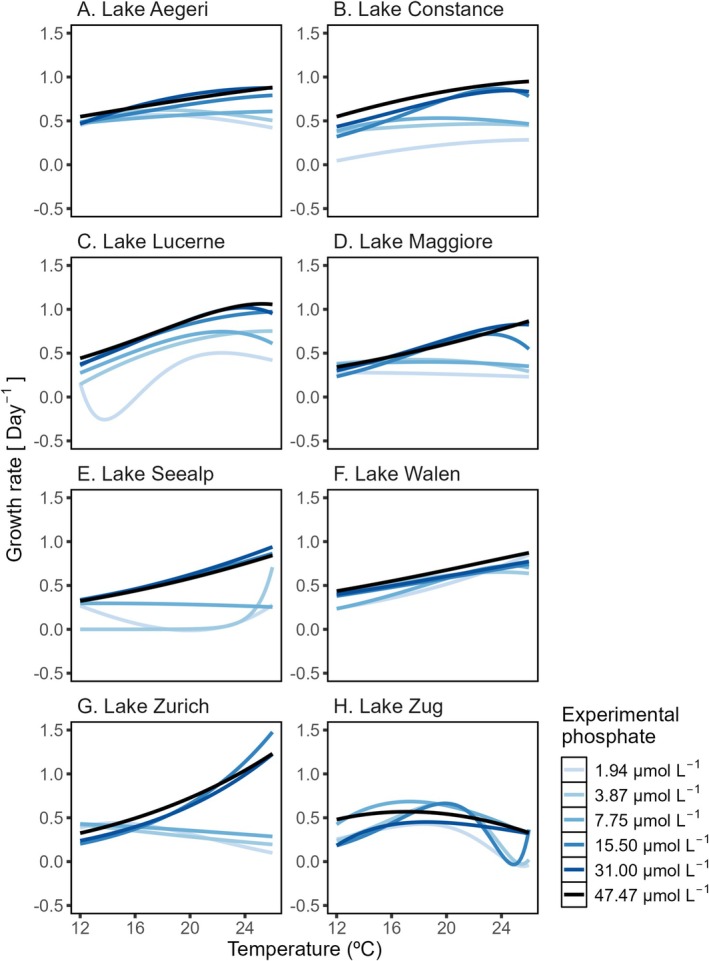
Modeled TPCs according to the modified Norberg function (Thomas et al. 2012) from the estimated growth rates of eight strains (A–H) across different experimental phosphorus concentrations. The TPCs show the relationship between intrinsic growth rate on the y‐axis and experimental temperature on the x‐axis for eight strains. Each curve was fitted to 24 data points (6 temperatures × 4 replicates); although in rare cases, some replicates had growth rates below the detection limit and were thus omitted (see Appendix S1).

Strains generally grew faster at higher temperatures (Figure 2). The TPCs in our experiment rarely captured a thermal optimum (*T*_opt_), indicating that the optimal temperature for growth was higher than the highest experimental temperature (Figure 2). The rate of increase of growth with temperature and the shapes of the TPCs depended both on the strain identity and phosphorus concentrations (Figure 2). At high phosphorus levels (47.47, 31.00 and 15.50 μmol·L^−1^), all strains except the one from Lake Zug grew faster as the temperature increased (Figure 2H); whereas at lower phosphorus levels (7.75, 3.87 and 1.94 μmol·L^−1^), strains from Lakes Aegeri, Maggiore and Zurich had relatively flat TPCs (Figure 2A,D,G).

**FIGURE 3:**
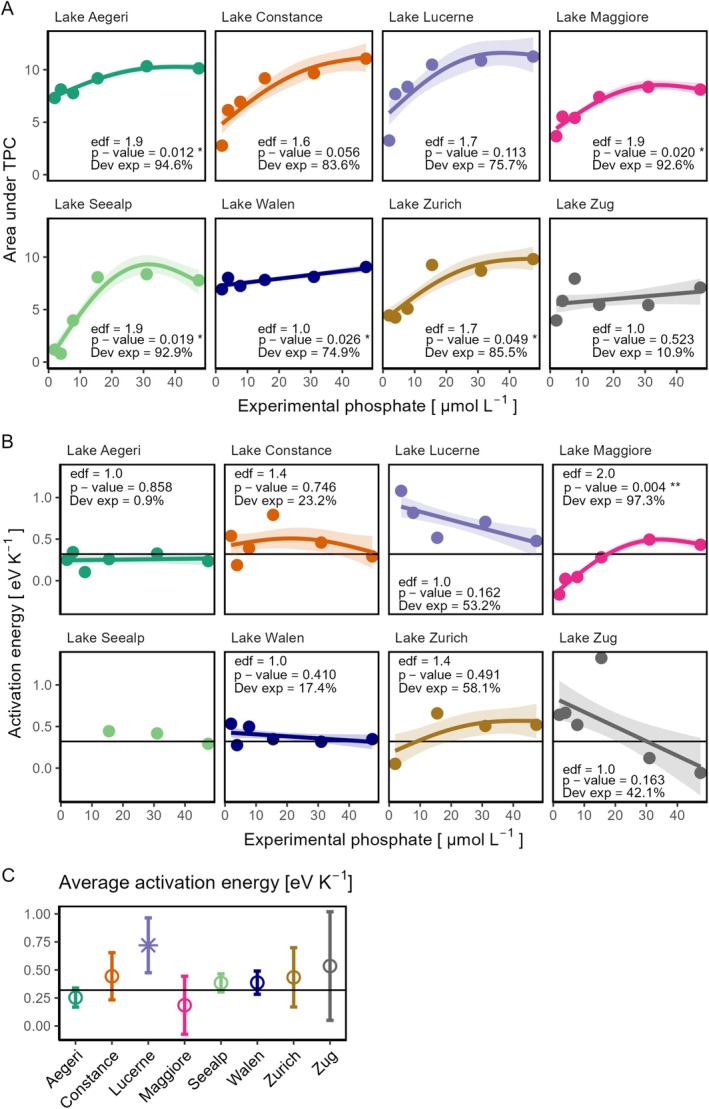
(A) Areas under the temperature performance curves (AUTPC) for all strains across different experimental phosphate concentrations. (B) Activation energy of growth rate for all strains across different experimental phosphorus concentrations. Colored solid lines are predictions from generalized additive models (GAMs), and shaded areas indicate the standard error of GAMs. The activation energy of the strain from Lake Seealp could be calculated at only three experimental phosphate levels; therefore, a GAM was not fitted for this strain. (C) The average activation energy of each strain across all experimental phosphorus levels. The error bars show the standard deviations. The horizontal lines in (B) and (C) mark the expectation of activation energy, 0.32 eV K^−1^. In (C), a star means that the average value is significantly different from 0.32 eV K^−1^ (one‐sample *t*‐test, *p*‐value < 0.05), whereas a hollow point indicates no significant difference.

The areas under the TPCs (AUTPC) increased with phosphorus for most strains in our study (Figure 3A). The strain from Lake Walen exhibited a linear increase in AUTPC with rising phosphorus levels (EDF = 1, *p*‐value < 0.05, Figure 3A), but this relationship was not statistically significant for the strain from Lake Zug (EDF = 1, *p*‐value > 0.05). All other strains showed non‐linear responses characterized by a rapid increase in AUTPC at low phosphorus levels, which then plateaued as phosphorus availability continued to rise (EDF values between 1.6 and 1.9, Figure 3A), but this pattern was only statistically significant for Lakes Aegeri, Maggiore, Seealp and Zurich. Higher AUTPCs in response to *P* availability indicate that strains had an overall higher performance across the thermal gradient when phosphate was abundant.

The activation energies of the population growth rate in the studied *Fragilaria* strains across all experimental phosphorus concentrations ranged from −0.16 to 1.32 eV (Figure S5B). For all but one strain, no statistically significant relationship was observed between activation energy and phosphorus availability (EDF values between 1 and 1.4, *p*‐values > 0.05, Figure 3B), supporting our hypothesis that phosphorus availability has no influence on the sensitivity of growth rate to temperature. Strains from Lakes Lucerne and Zug exhibited a slight decrease in *E*_a_ with increasing phosphorus, but this trend was not statistically significant. The strain from Lake Maggiore is the only one with a significant relationship between its *E*_a_ and the phosphorus gradient. Its *E*_a_ increased non‐linearly as phosphate levels rose, eventually saturating at 31.00 μmol·L^−1^ (*p*‐value < 0.01, EDF = 2.0, GAM explained 97.2% of the deviance, Figure 3B). The average *E*_a_ for each strain across all phosphorus levels was not significantly different from the expectation of 0.32 eV K^−1^, except for the strain from Lake Lucerne (Figure 3C). These findings showed that *E*_a_ was not affected by phosphorus availability, and there was little variability among strains.

### 3.2 Temperature Sensitivity of Monod Curves

**FIGURE 4:**
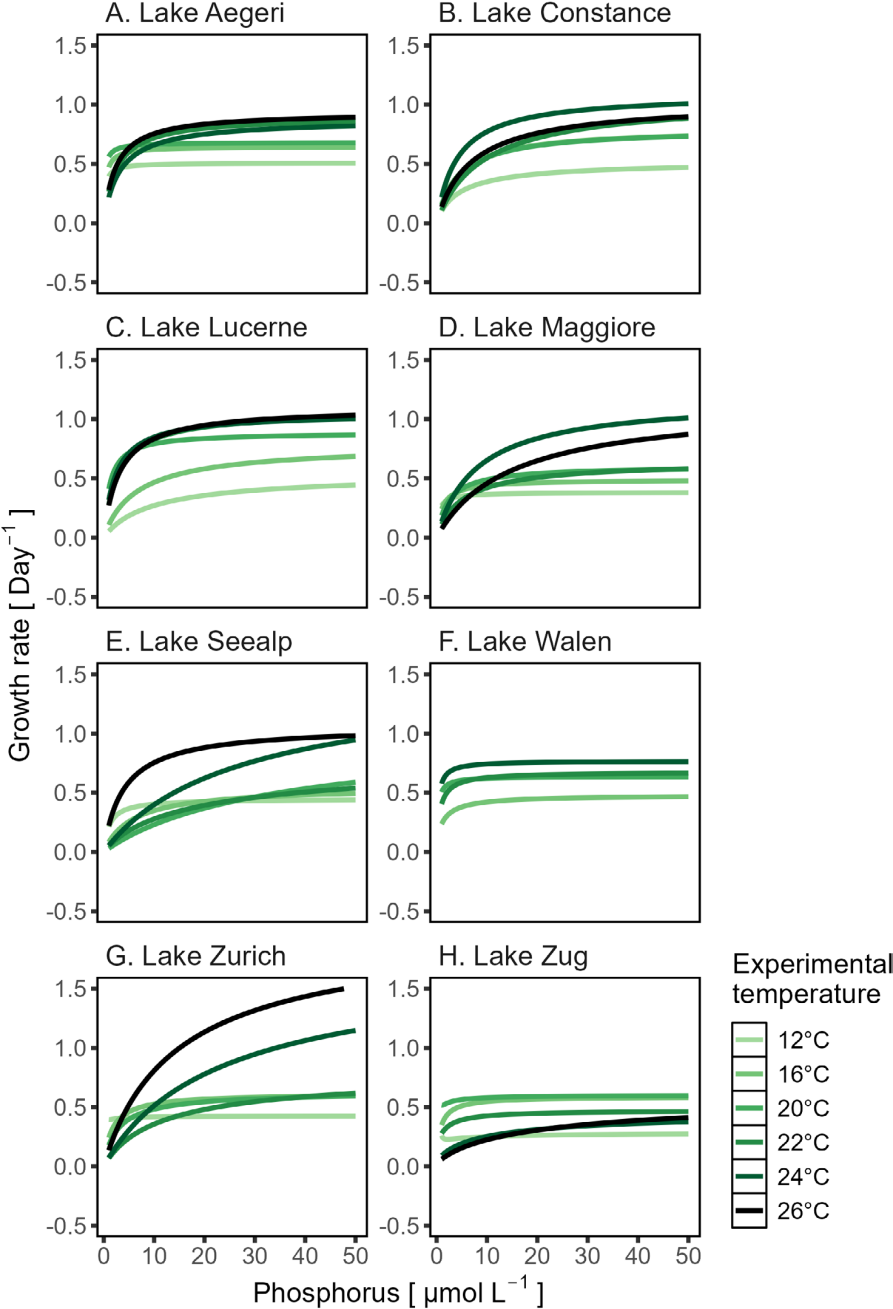
Monod curves fitted to growth rates of eight strains (A–H) across different experimental temperatures. The Monod curves show the relationship between intrinsic growth rate on the *y*‐axis and experimental phosphate concentration (μmol·L^−1^) on the *x*‐axis for each of the eight strains. Each curve was fitted to 24 data points (6 temperatures × 4 replicates); although in rare cases, some replicates had growth rates below the detection limit and were thus omitted (see Appendix S1 for the complete dataset).

At a given temperature, most strains grew faster with increasing phosphorus availability (Figure 4). Strains from lakes Aegeri, Constance, Lucerne, Maggiore, Seealp and Zurich (Figure 4A–E,G) had flatter curves at low temperatures (12°C and 16°C), indicating that the increase of phosphate concentration only weakly influenced growth rates in cold environments. In contrast, at high temperatures (22°C–26°C), their growth rate increased rapidly with phosphorus availability, resulting in steeper Monod curves. Monod curves of strains from lakes Walen (Figure 4F) and Zug (Figure 4H) were mostly flat, with only slight variation in maximum growth rate across temperatures, showing that their growth rates changed with temperature but responded little to phosphate in our experiments.

**FIGURE 5:**
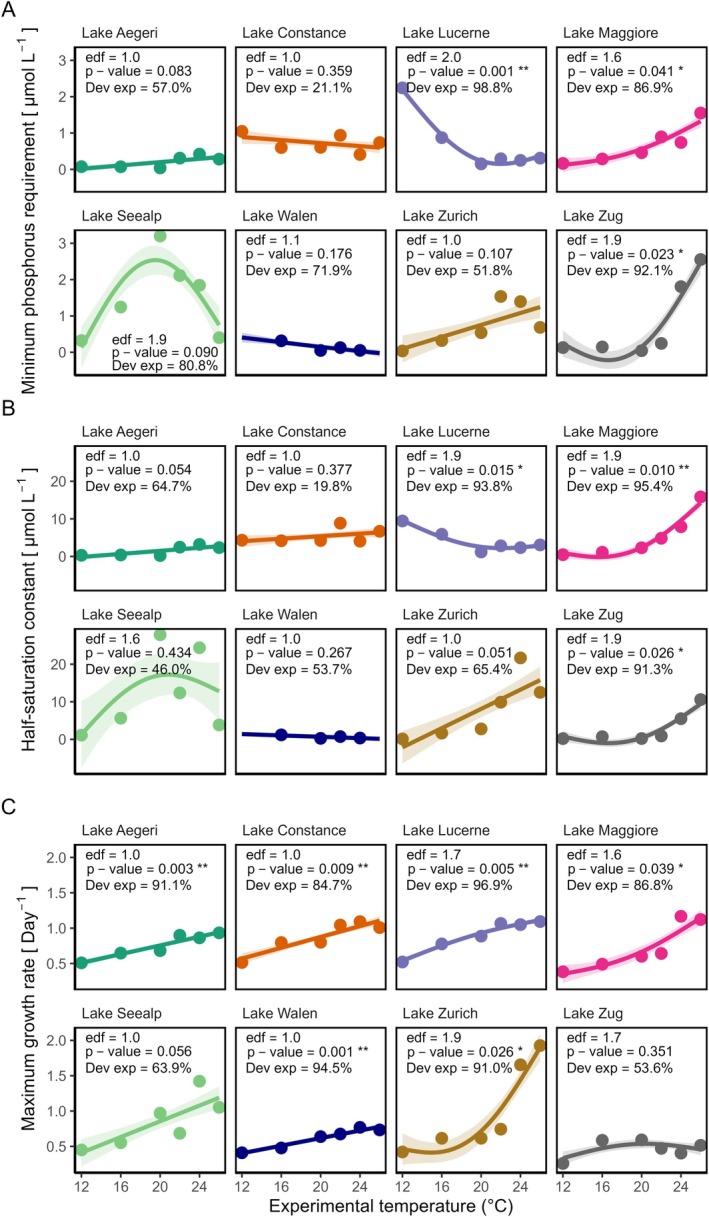
(A) Minimum phosphorus requirements (P*), (B) half‐saturation constant (*K*_s_), and (C) Maximum growth rate (*μ*_max_) of eight strains across different experimental temperatures. P* was calculated using the coefficients of estimated *K*_s_ and *μ*_max_ values from the fitted Monod curves. Both *K*_s_ and *μ*_max_ were estimated from growth rates using the Monod equation fitted through non‐linear least squares. Data points represent calculated values, whereas solid lines are predictions from generalized additive models (GAMs). Shades indicate the standard error of GAMs. Two Monod curves of the strain from Lake Walen had negative values of P* and *K*_s_, which are not biologically meaningful. Those data points were removed from (A) and (B).

The P* values ranged from 0.04 to 3.20 μmol·L^−1^ across all strains and experimental temperatures (Figure 5A, Figure S6A). The strains from lakes Lucerne and Zug displayed a U‐shaped relationship between P* and experimental temperature (*p*‐value < 0.05, and GAMs explained > 90% of the variation, Figure 5A), aligning with the expected relationship between R* and temperature. For these two strains, P* reached the lowest at 20°C and 17°C, respectively (Figure 5A). Furthermore, the GAM for the strain from Lake Maggiore was also significant, explaining 86.9% of deviance, and had an EDF of 1.6. Within the experimental temperature range, the P* of this strain increased continuously with temperature. This did not contradict the U‐shape expectation because we might not have observed the full curvature over our experimental temperature gradient. The GAMs of the remaining five strains showed either a linear relationship or a non‐linear relationship with a hump‐shape, but they were not significant (*p*‐value > 0.05, explaining between 21% and 80% of the variance), indicating that there was no association between P* and experimental temperature (Figure 5A). The patterns of *K*_s_ of each strain closely matched those of P* (Figure 5B).

The *μ*_max_ of all strains (except the one from Lake Zug) increased with experimental temperature (six out of seven with a *p*‐value < 0.05, Figure 5C). Although the *μ*_max_ increased consistently with temperature among strains, the linearity of the relationship varied, with four linear relationships (EDF = 1.0) and three near non‐linear (EDF values between 1.6 and 1.9, Figure 5C).

### 3.3 Local Adaptation of Thermal and Resource‐Use Traits

**FIGURE 6:**
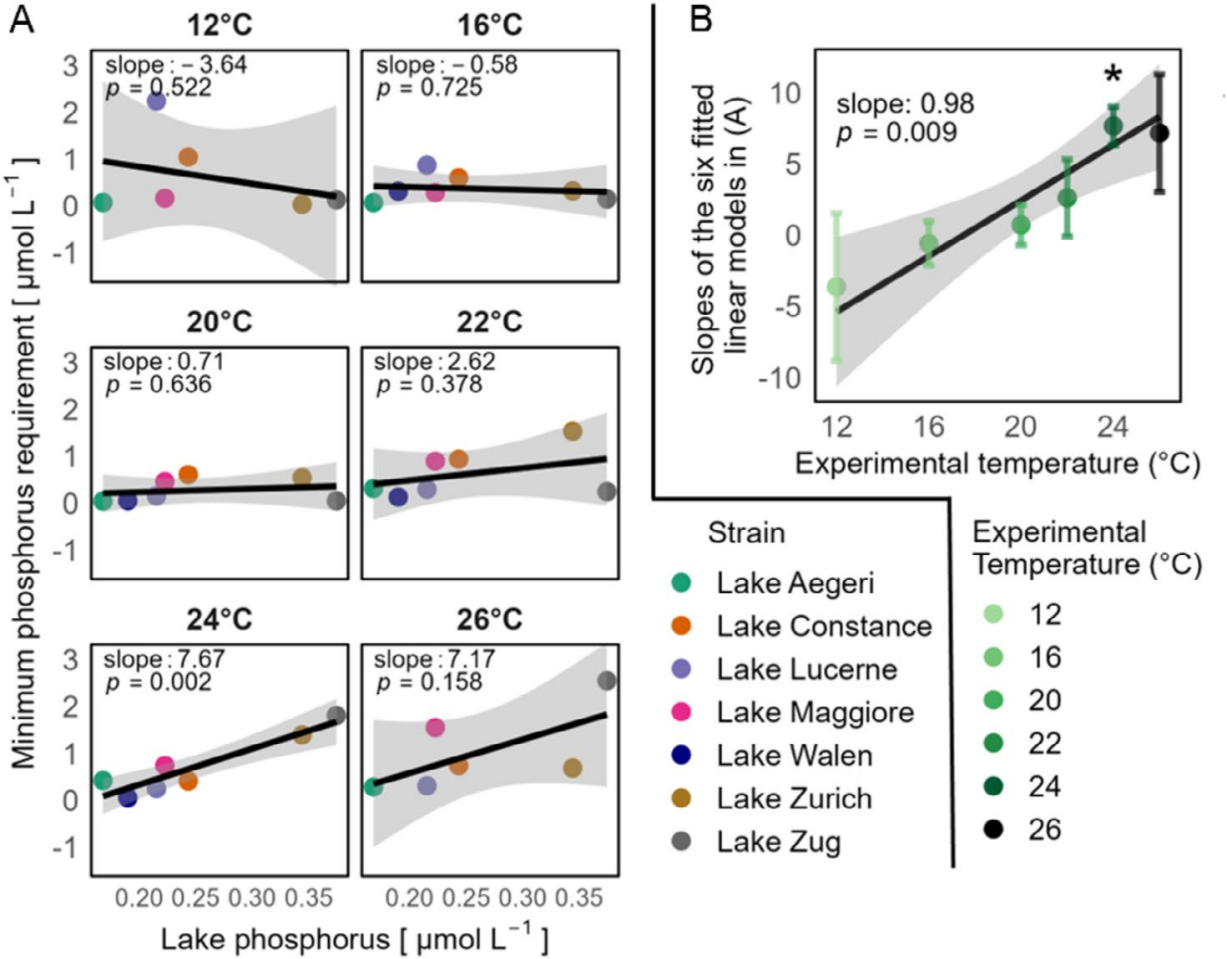
(A) Temperature‐dependent relationship between P* and lake phosphorus concentrations for eight diatom strains. The figure shows the relationship between P* of strains and the average total phosphorus in the last 5 years from July to September in the corresponding lakes across different experimental temperatures. Black lines are fitted linear regressions, whereas gray shades indicate the 95% confidence intervals (CI) of the fitted relationships. Slopes and *p*‐values of the linear regressions are shown in each facet. (B) Linear regression of the slopes from (A) against experimental temperature. Slopes from (A) were weighted by the inverse of their squared standard errors (1/SE^2^) to account for differences in estimation precision. Error bars show the standard error of each slope estimate from the individual regressions in (A). The gray area represents the 95% CI of the fitted relationship. An asterisk above a data point indicates that the corresponding linear regression in (A) has a *p*‐value lower than 0.05.

For most traits, we did not observe strong relationships between the traits of the strains and the environmental conditions of the lakes from which these strains were isolated (Figure S7). However, we report a notable exception with the minimum phosphorus requirements (P*). The relationship of P* of the different strains to the phosphorus content of the lake from which they were isolated tended not to be statistically significant (but see the relationship at 24°C, Figure 6A), but when we investigated how the slopes of these relationships varied with the experimental temperature under which they were measured, we observed a positive relationship (*p*‐value < 0.01, Figure 6B). This positive association was mainly due to the increase in P* of the strains from phosphorus‐rich lakes when measured under warmer temperatures. By contrast, the P* of the strains from lakes with lower phosphorus concentrations was little affected by experimental temperature (Figure 6A). This indicates that the P* of strains from phosphorus‐rich lakes was more sensitive to temperature compared to those from lakes with lower phosphorus concentrations.

## 4 Discussion

Our results describe the interactive effects of temperature and phosphorus availability on the growth rates of multiple strains of a single phytoplankton species, the freshwater diatom *F. crotonensis* . Both higher phosphorus concentrations and higher temperatures (within the range of our experiment) resulted in increased growth rates. The dependencies of TPC parameters on phosphorus, as well as of Monod curve parameters on temperature, suggest that the strength of these interactive effects of temperature and resource availability on growth may depend on the environment from which the strains were isolated, providing some initial support for a role of local adaptation. Our results also suggest that populations of *F. crotonensis* from high‐phosphorus lakes tend to display increases in phosphorus requirements under warming conditions. In turn, populations from lakes with higher phosphorus availability may suffer greater increases in nutrient stress than populations from low‐phosphorus lakes under future conditions of combined warming and resource limitation.

### 4.1 Phosphorus Dependence of Thermal Traits

Although phosphorus positively influenced overall performance across the thermal gradient, as measured by the AUTPC, the activation energy of population growth rate remained largely unaffected by phosphorus availability. For most strains, AUTPC followed a saturating pattern with increasing experimental phosphate, consistent with the metabolic meltdown hypothesis (Huey and Kingsolver 2019), which predicts a contraction of the TPC under resource limitation. On the other hand, only one out of eight strains displayed a significant relationship between phosphorus availability and activation energy, indicating that there was no strong support for an effect of experimental phosphate on population growth rate activation energy. This confirms the result of Weber de Melo et al. (2025), who did not detect a significant difference in the activation energy of growth rate among the three levels of phosphorus in green algae and cyanobacteria. Taken together, these results suggest that although higher phosphorus availability enhances overall growth, it does not alter the sensitivity of population growth rates to temperature.

### 4.2 Temperature Dependence of Resource‐Use Traits

Previous studies suggest that the relationship of minimum resource requirements against temperature is U‐shaped (Tilman 2004; Thomas et al. 2017). One of the reasons is that warming reduces the costs of overall metabolism for intermediate temperatures and increases them again toward the warmer end of the thermal niche (Van de Waal and Litchman 2020; Thomas et al. 2017). Three of eight strains showed this U‐shaped relationship between P* and experimental temperature (strains from lakes Lucerne, Maggiore and Zug). Only a small number of studies reported the P* and *K*_s_ of *F. crotonensis,* and in general, the values were very low (Table 3). In our study, the values of P* and *K*_s_ were sometimes close to those in previous reports, especially for the strains from low‐phosphorus lakes (such as lakes Aegeri and Walen), or under low experimental temperatures. Previous low values of P* and *K*_s_ have also been estimated using cold temperatures or strains isolated from low‐phosphorus lakes (Table 3). Tilman (1981) and van Donk and Kilham (1990) used relatively low experimental temperatures, and Michel et al. (2006) collected the studied diatom from lakes with very low phosphorus availability. Variations in experimental design and methodology may also lead to different P* and *K*_s_ values. For instance, in Tilman's (1981) experiment, cultures underwent a phosphorus acclimation before the growth experiment, in which cultures were inoculated into zero phosphorus medium (with potential phosphorus carry over) for 4 weeks, whereas we acclimated our strains for 3 days, after rinsing cultures and replacing the medium with our target phosphate concentrations. van Donk and Kilham (1990) directly measured *μ*_max_ using separate continuous batch culture experiments rather than estimating it through Monod function modeling on the basis of growth experiments. However, despite the methodological differences, their measured *μ*_max_ values and trends matched our findings. The in situ bioassays performed by Michel et al. (2006) could not rule out the impact from other species, since multiple species were grown together; the growth rate was calculated on the basis of only the initial and final cell densities, which may not have accurately captured the maximum slope of the exponential growth phase. New methods and technological advances allowed us to minimize the effect of factors other than temperature and phosphorus on the growth of diatoms, while investigating multiple strains and crossed environmental gradients at the same time—something which was not possible in these prior studies.

**TABLE 3:** Comparison between previous studies measuring resource‐use traits of *F. crotonensis* and this study.

Sampling site	Lake phosphorus measurement	Sampling time	Experiment	Experimental temperature (°C)	P* (μmol·L^−1^)	*K*_s_ (μmol·L^−1^)	*μ*_max_ (Day^−1^)	Study
Lake Michigan, USA	0.23 μmol·L^−1^, total phosphorus, measured in 1980, Dove and Chapra (2015)	During the spring diatom bloom	Single‐species batch culture growth lab experiment	~12	0.005 (0.002–0.008)	0.011 (0–0.024)	0.80 (0.72–0.88)	Tilman (1981)
Lake Maarsseveen, the Netherlands	0.65 μmol·L^−1^, total phosphorus, measured in 1980, van Donk (1987)	2 months before the experiment (exact time n/a)	Single‐species batch culture growth lab experiment	5, 10, 15, and 20 (showing data at 15)	0.025	0.050 (0.032–0.070)	0.60 (0.56–0.64)	van Donk and Kilham (1990)
Alpine lakes around the Beartooth Mountain Range, USA	Below detection 0.02 μmol·L^−1^, soluble reactive phosphorus (SRP), 2003, Saros et al. (2003)	n/a	In situ (Beauty Lake, Park Co., WY) batch culture (mixed species) bioassays	20	n/a	0.0008 (0.065)	0.37 (0.004)	Michel et al. (2006)
Eight Swiss alpine and plain lakes	Table S5	End of summer	Single‐species batch culture growth lab experiment	Six levels, from 12 to 26	Figure 5A, Figure S5A	Figure 5B, Figure S5B	Figure 5C, Figure S5C	This study

*Note:* The first three studies did not measure the phosphorus concentration in the lakes they sampled, so we took measurements from other literature that are as close as possible in terms of time and location. P*, *K*_s_ and *μ*_max_, inside the parentheses are the 95% confidence intervals (Tilman 1981; van Donk and Kilham 1990) or standard error (Michel et al. 2006).

### 4.3 Association Between Traits and Environmental Variables

Our results showed that the lake's total phosphorus concentration is associated with the P* of the strain at 24°C. Specifically, strains from more phosphorus‐rich lakes had higher P*, confirming our initial hypothesis. In addition, lake phosphorus concentration also influences the sensitivity of strains' P* to temperature. The relationship between lake phosphorus concentration and P* changed from no relationship to a positive relationship as the experimental temperatures increased. This occurred because the P* of strains from lakes with low phosphorus concentrations remained relatively constant across different temperatures in the experiment, whereas the P*s of strains from high phosphorus lakes were more plastic, and increased in response to increasing temperatures. This suggests that as temperatures rise, populations from phosphorus‐rich environments may face intensified resource limitations because of increases in their P*, making their survival and adaptation to future warmer and more nutrient‐poor conditions less likely. However, we were unable to see strong correlations in environmental conditions with other resource‐use traits and thermal traits. A higher number of strains and a broader temperature or phosphorus gradient among lakes might help to reveal more patterns.

### 4.4 Limitations and Future Directions

Our study has offered initial insights into the intraspecific diversity of the thermal and resource‐use traits of phytoplankton and pointed out its potential implications in the adaptation of phytoplankton to environmental change. We have recognized the importance of considering the interactive effects of temperature and resource availability, as well as the intraspecific diversity. However, some limitations remain. First, using one strain per lake may restrict our ability to capture within‐lake diversity. Additionally, the isolation process can introduce a strong bottleneck effect, potentially resulting in many isolates from the same lake being genetically identical. Future work examining within‐lake variation across seasons or years would be valuable, as such approaches may increase the likelihood of detecting distinct genotypes from the same lake. Besides, since the *T*_opt_ might fall outside our experimental range, we could not confirm whether strains coming from warmer lakes would have higher *T*_opt_ values. Future studies could include more data points at high temperatures, since the TPC of phytoplankton drops sharply after the *T*_opt_ (Eppley 1972). A further point is that, for some strains, the change of P* with our experimental temperatures was minimal and the GAM predictions were almost flat. Both suggest the need to expand the temperature range to better test the expected trends. In addition, some strains had nearly flat Monod curves at all experimental temperatures, indicating saturated growth rates. Lower phosphate concentrations in future experiments could help better characterize phosphorus‐limited growth. More general conclusions about the importance of intraspecific variation to these traits are expected to be made from future experiments with more strains and more complete thermal and resource trait characterizations. Other species belonging to functional groups beyond diatoms, such as chlorophyta, cyanobacteria and chrysophytes, should be studied to quantify the natural intraspecific diversity of these traits.

## Author Contributions

**Li Zhao:** conceptualization (equal), data curation (equal), formal analysis (equal), investigation (equal), methodology (equal), project administration (equal), software (equal), visualization (equal), writing – original draft (equal), writing – review and editing (equal). **Divina Ryf:** conceptualization (equal), data curation (equal), formal analysis (equal), investigation (equal), methodology (equal), project administration (equal), software (equal), visualization (equal), writing – original draft (equal), writing – review and editing (equal). **Sarah Levasseur:** conceptualization (equal), investigation (equal), methodology (equal), software (equal), writing – review and editing (equal). **Raphaël Bossart:** formal analysis (equal), investigation (equal), methodology (equal), writing – review and editing (equal). **Marta Reyes:** investigation (equal), methodology (equal), writing – review and editing (equal). **Frank Pennekamp:** conceptualization (equal), supervision (equal), writing – review and editing (equal). **Jukka Jokela:** conceptualization (equal), supervision (equal), writing – review and editing (equal). **Anita Narwani:** conceptualization (equal), formal analysis (equal), funding acquisition (equal), supervision (equal), writing – original draft (equal), writing – review and editing (equal). **Vanessa Weber de Melo:** conceptualization (equal), formal analysis (equal), investigation (equal), methodology (equal), project administration (equal), software (equal), supervision (equal), writing – original draft (equal), writing – review and editing (equal).

## Conflicts of Interest

The authors declare no conflicts of interest.

## Supporting information


**Appendix S1:** ece372427‐sup‐0001‐AppendixS1.pdf.

## Data Availability

All data and related Supporting Information are available on Dryad (https://doi.org/10.5061/dryad.83bk3jb58). Sequencing data have been deposited on GenBank and can be accessed with the following accession numbers: PV932063–PV932070 (18S sequences) and PV941022–PV941029 (rbcL sequences).
