# Supplementary material for: Evidence of Local Adaptation in a Freshwater Diatom Indicates Higher Sensitivity to Nutrient Limitation as Water Temperature Rises
Source: Ecol Evol. 2025 Nov 9;15(11):e72427. doi: 10.1002/ece3.72427 (PMC12597979; doi:10.1002/ece3.72427)
Supplement: Supplementary file 1 — Appendix S1: ece372427‐sup‐0001‐AppendixS1.pdf. [file ECE3-15-e72427-s001.pdf]

Evidence of local adaptation in a freshwater diatom indicates higher sensitivity to nutrient limitation as water temperature rises

Supplementary Figures and Tables

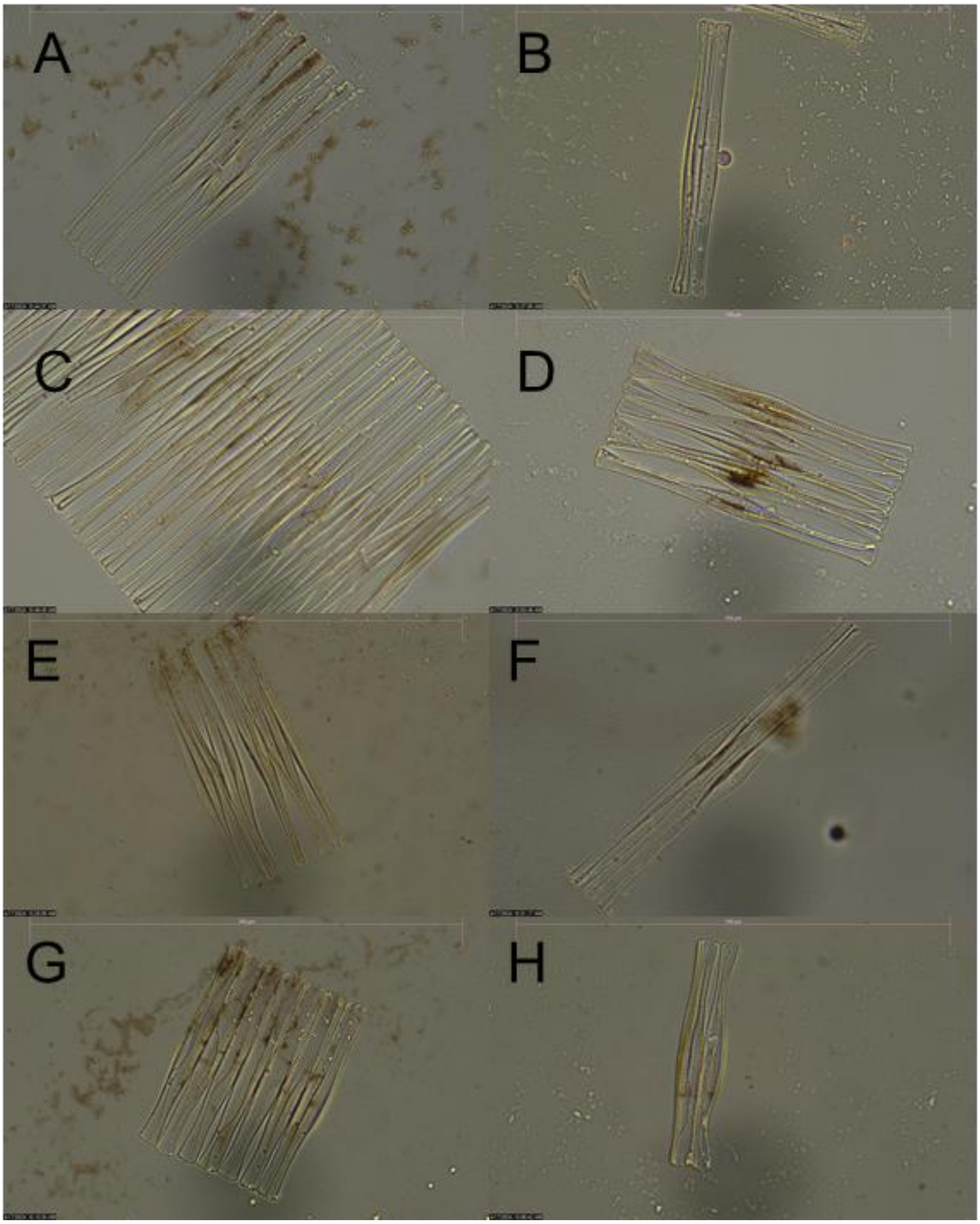

Figure S1. Images of mounted *F. crotonensis* cells under 100× magnification generated by light microscopy. All images are shown at the same magnification. Scale bars (on the top of each image) represent 100 µm. Letters A to H represent strains from lakes Aegeri, Constance, Lucerne, Maggiore, Seealp, Walen, Zurich and Zug.

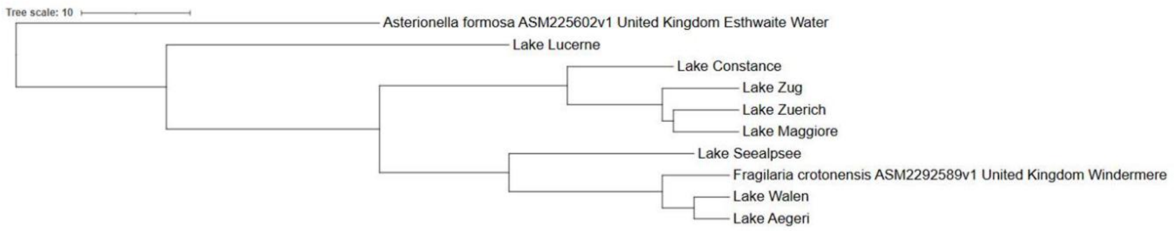

Figure S2. Distance tree generated based on ITS region, illustrating the relationships among the eight isolated strains. Two outgroup taxa were included: the diatom *Asterionella formosa* and a reference sequence of *Fragilaria crotonensis*. GenBank accession numbers for these sequences are provided alongside species names in the figure.

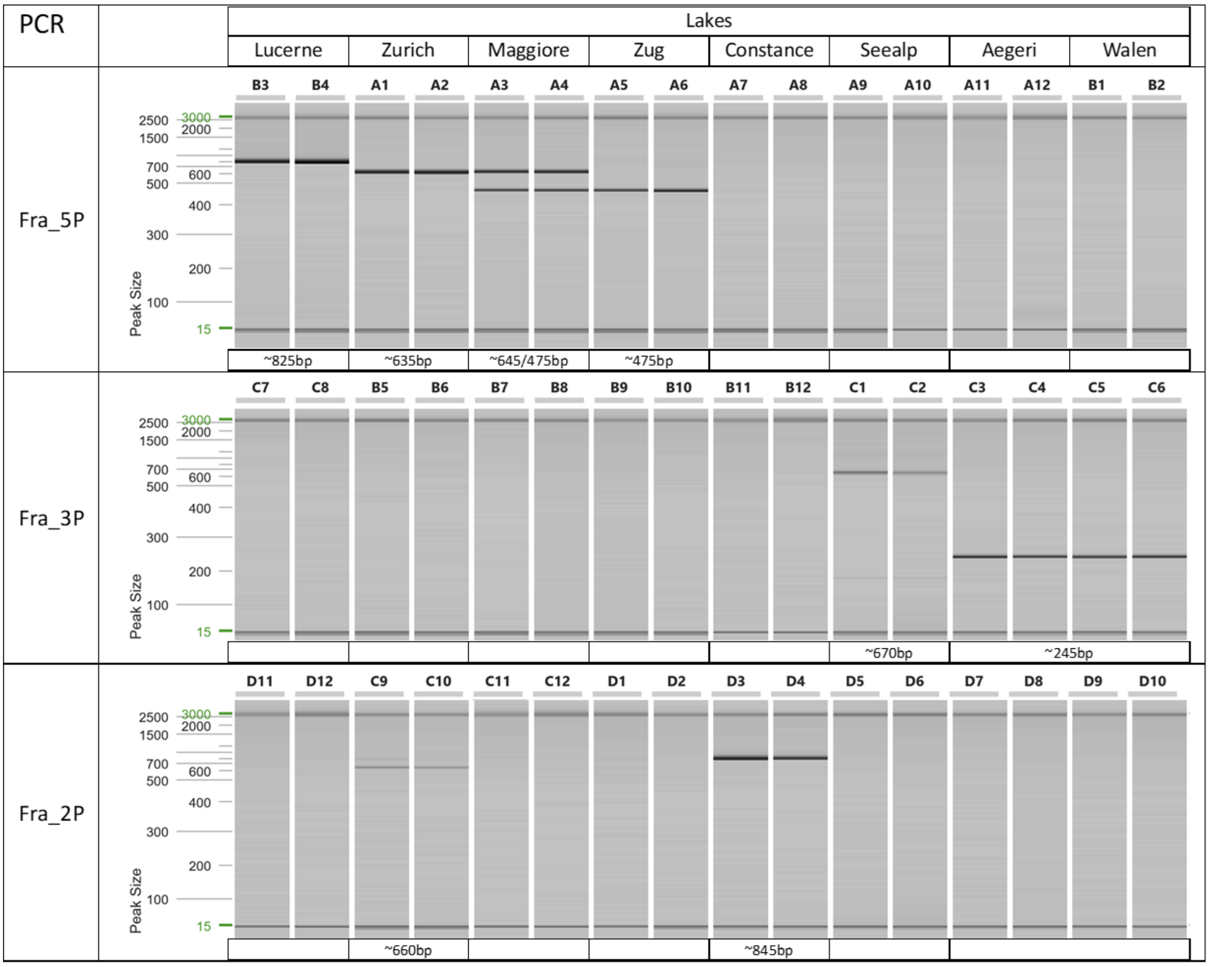

Figure S3. Gel electrophoresis of amplified ITS gene with three multiplex PCR across the eight studied lakes. Each sample corresponds to one isolate of a lake, and lake names are shown on top of the image. Two isolates of each lake were tested. Multiplex PCR Fra_5P includes primer pairs 1, 2 and 3, Fra_3P includes primer pairs 4 and 5 and Fra_2P includes primer pair 6 (see Table S2 for more details).

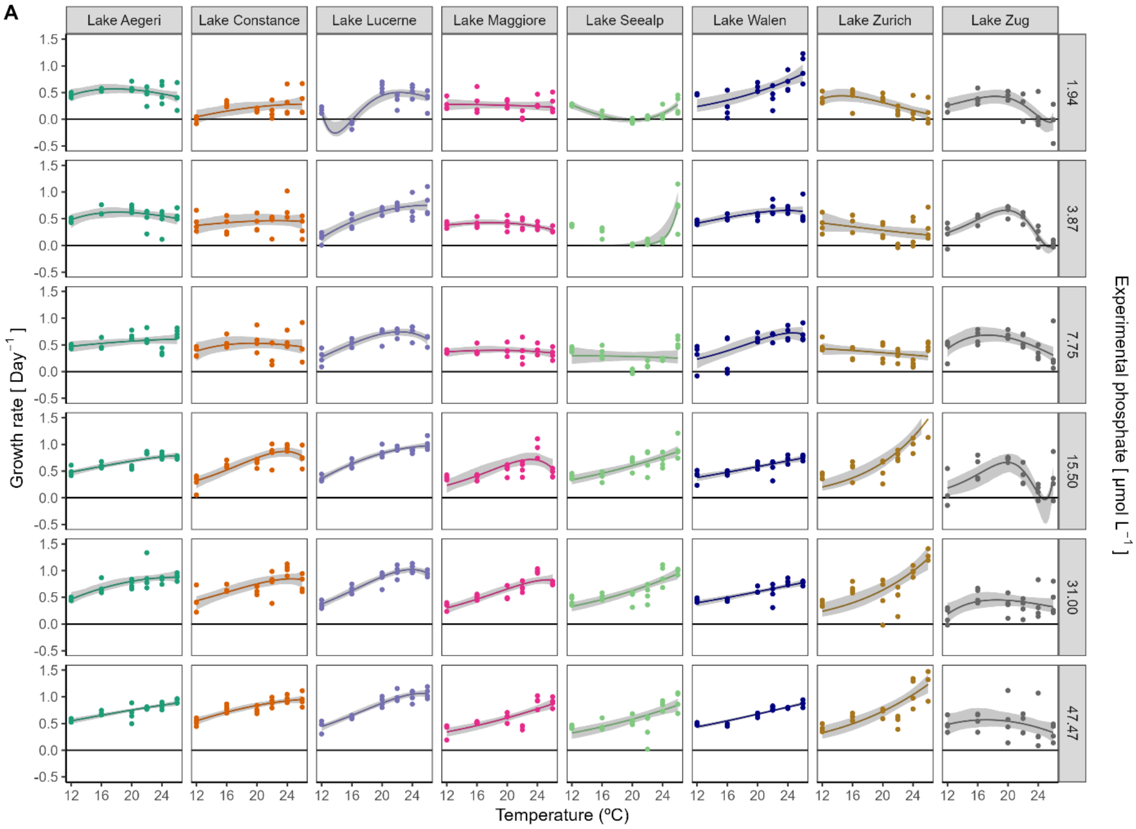

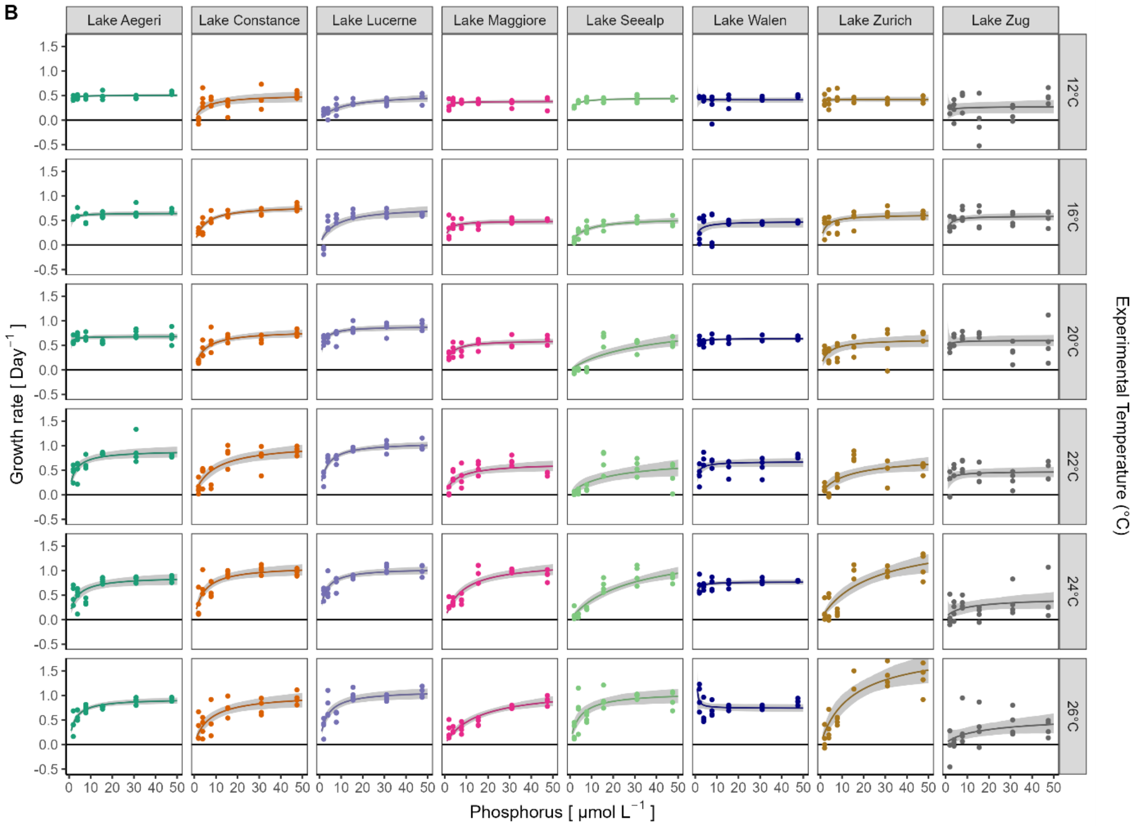

Figure S4. TPCs (A) and Monod (B) curves with data points, as expansions to Figure 2 and 4. Each column represents one strain and each row is one level of temperature or phosphorus in the experiment.

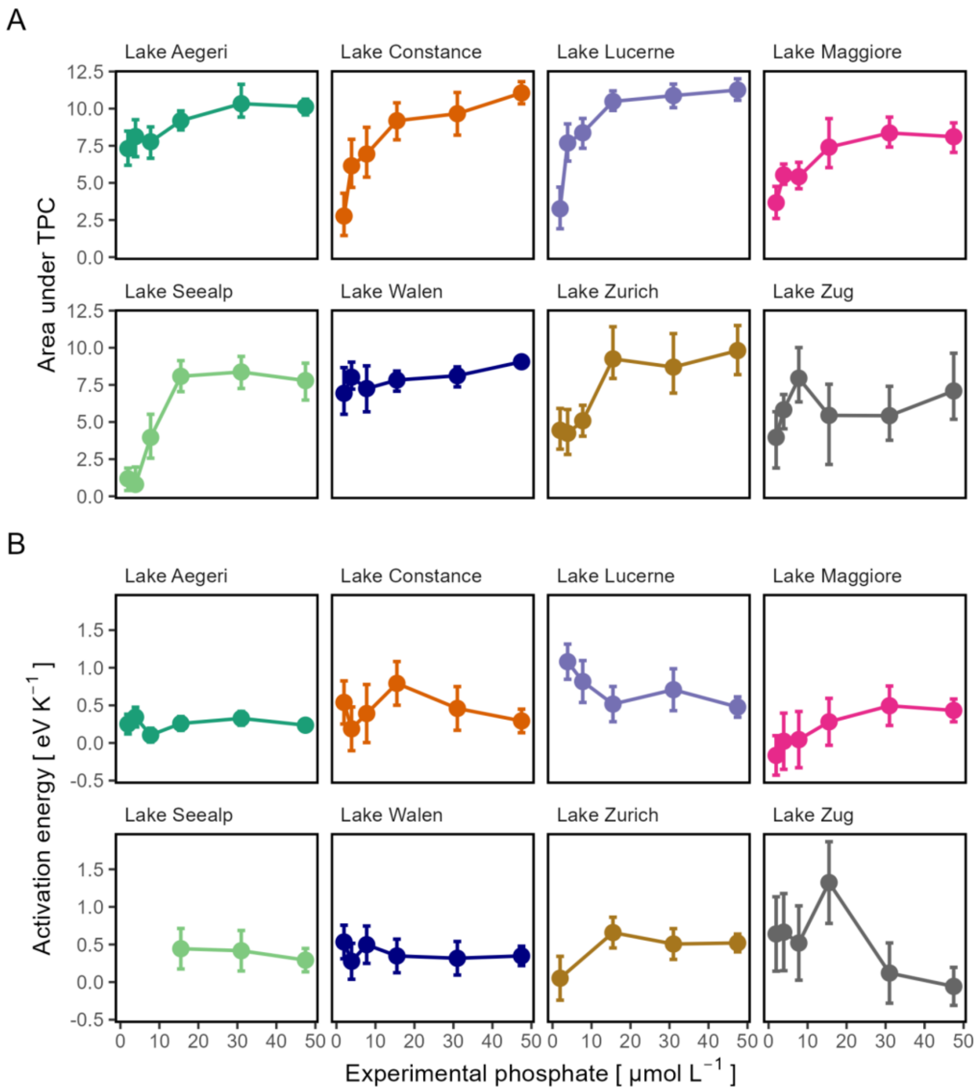

Figure S5. (A) Calculated areas under the modelled temperature performance curves (AUTPC) for all strains across different experimental phosphorus concentrations. When modelling TPCs, we yielded the upper and lower bounds of the 95% confidence interval (CI) through bootstrapping. The error bars represent, for each strain at each experimental phosphate level, the range between the area under the upper bound of the CI and the area under the lower bound of the CI. Those areas are calculated with the same method as AUTPC. (B) Estimated activation energy of growth rate for all strains across different experimental phosphorus concentrations. Error bars indicate standard deviations.

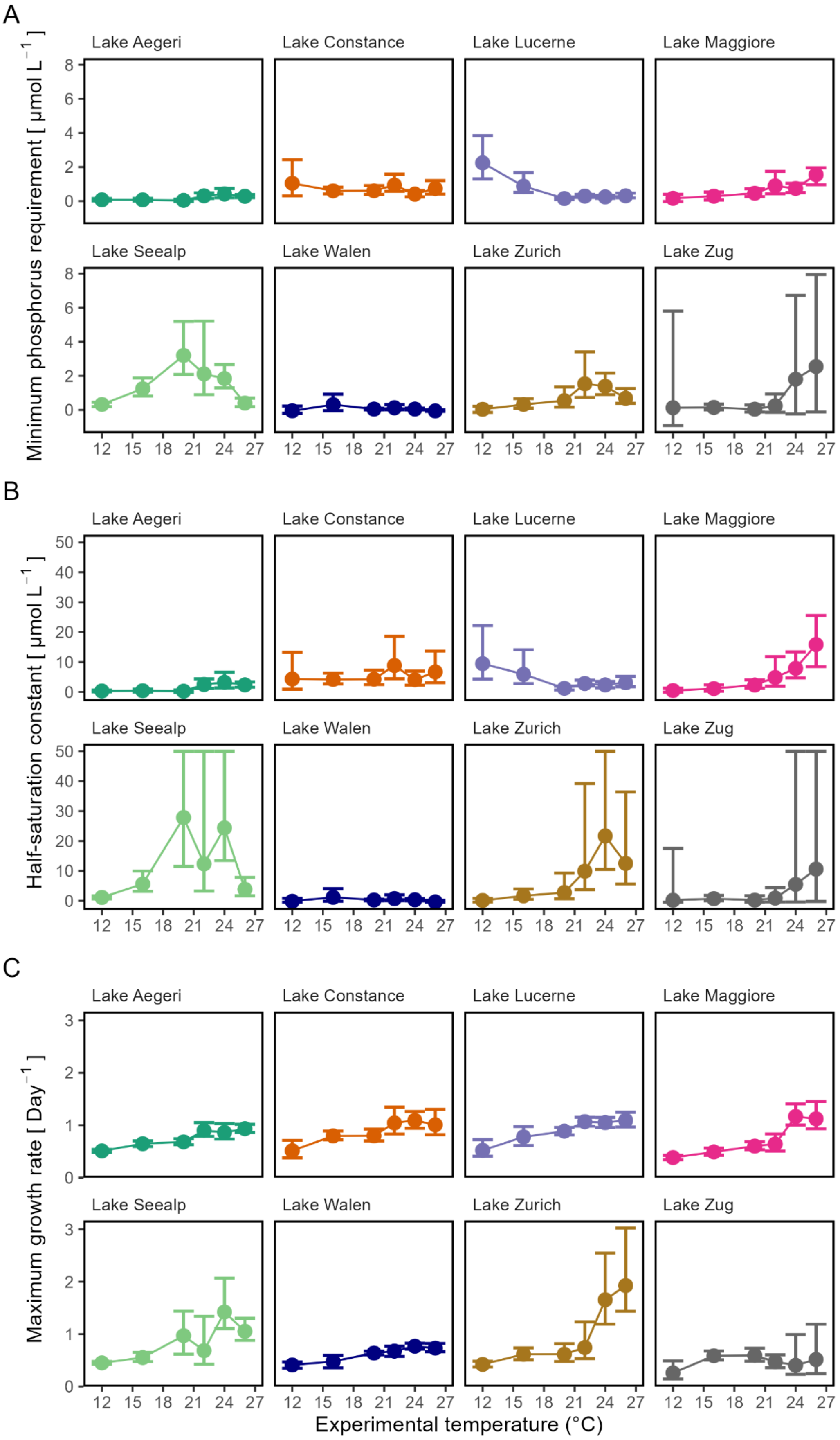

A
Lake Aegeri
Lake Constance
Lake Lucerne
Lake Maggiore
Lake Seealp
Lake Walen
Lake Zurich
Lake Zug
Minimum phosphorus requirement [μmol L⁻¹]
B
Half-saturation constant [μmol L⁻¹]
C
Maximum growth rate [Day⁻¹]
Experimental temperature (°C)

Figure S6. (A) Minimum phosphorus requirements (*P**), (B) half-saturation constant (K_s), and (C) Maximum growth rate (μ_{max}) of eight strains across different experimental temperatures. *P** was calculated using the coefficients of estimated K_s and μ_{max} values from the fitted Monod curves. Both K_s and μ_{max} were estimated from growth rates using the Monod equation fitted through nonlinear least squares. Error bars indicate 95% confidence intervals from bootstrapping.

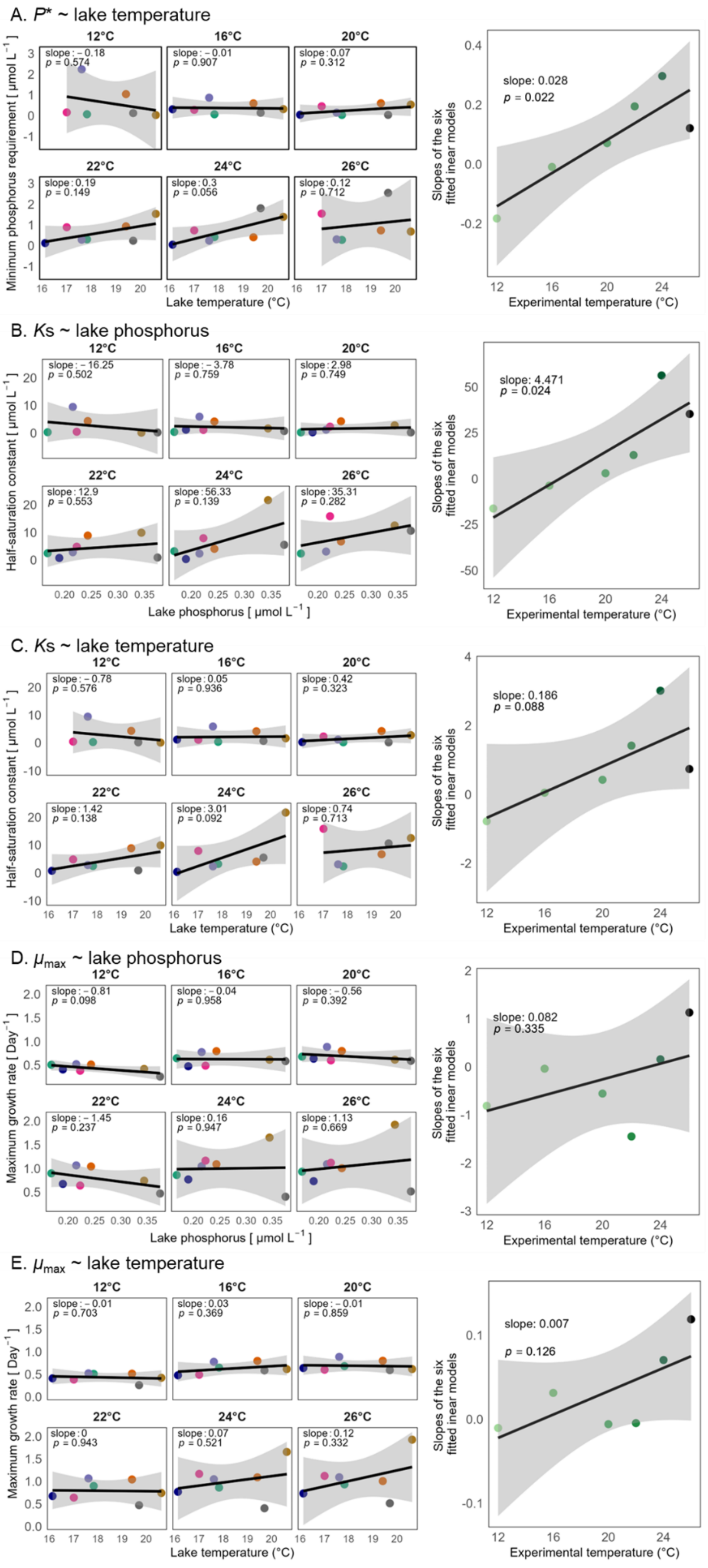
A. P* ~ lake temperature
12°C
16°C
20°C
22°C
24°C
26°C
slope : – 0.18
p = 0.574
slope : – 0.01
p = 0.907
slope : 0.07
p = 0.312
slope : 0.19
p = 0.149
slope : 0.3
p = 0.056
slope : 0.12
p = 0.712
Minimum phosphorus requirement [µmol L⁻¹]
Lake temperature (°C)
slope: 0.028
p = 0.022
Slopes of the six fitted inear models
Experimental temperature (°C)
B. Ks ~ lake phosphorus
slope : – 16.25
p = 0.502
slope : – 3.78
p = 0.759
slope : 2.98
p = 0.749
slope : 12.9
p = 0.553
slope : 56.33
p = 0.139
slope : 35.31
p = 0.282
Half-saturation constant [µmol L⁻¹]
Lake phosphorus [µmol L⁻¹]
slope: 4.471
p = 0.024
C. Ks ~ lake temperature
slope : – 0.78
p = 0.576
slope : 0.05
p = 0.936
slope : 0.42
p = 0.323
slope : 1.42
p = 0.138
slope : 3.01
p = 0.092
slope : 0.74
p = 0.713
slope: 0.186
p = 0.088
D. µmax ~ lake phosphorus
slope : – 0.81
p = 0.098
slope : – 0.04
p = 0.958
slope : – 0.56
p = 0.392
slope : – 1.45
p = 0.237
slope : 0.16
p = 0.947
slope : 1.13
p = 0.669
Maximum growth rate [Day⁻¹]
slope: 0.082
p = 0.335
E. µmax ~ lake temperature
slope : – 0.01
p = 0.703
slope : 0.03
p = 0.369
slope : – 0.01
p = 0.859
slope : 0
p = 0.943
slope : 0.07
p = 0.521
slope : 0.12
p = 0.332
slope: 0.007
p = 0.126

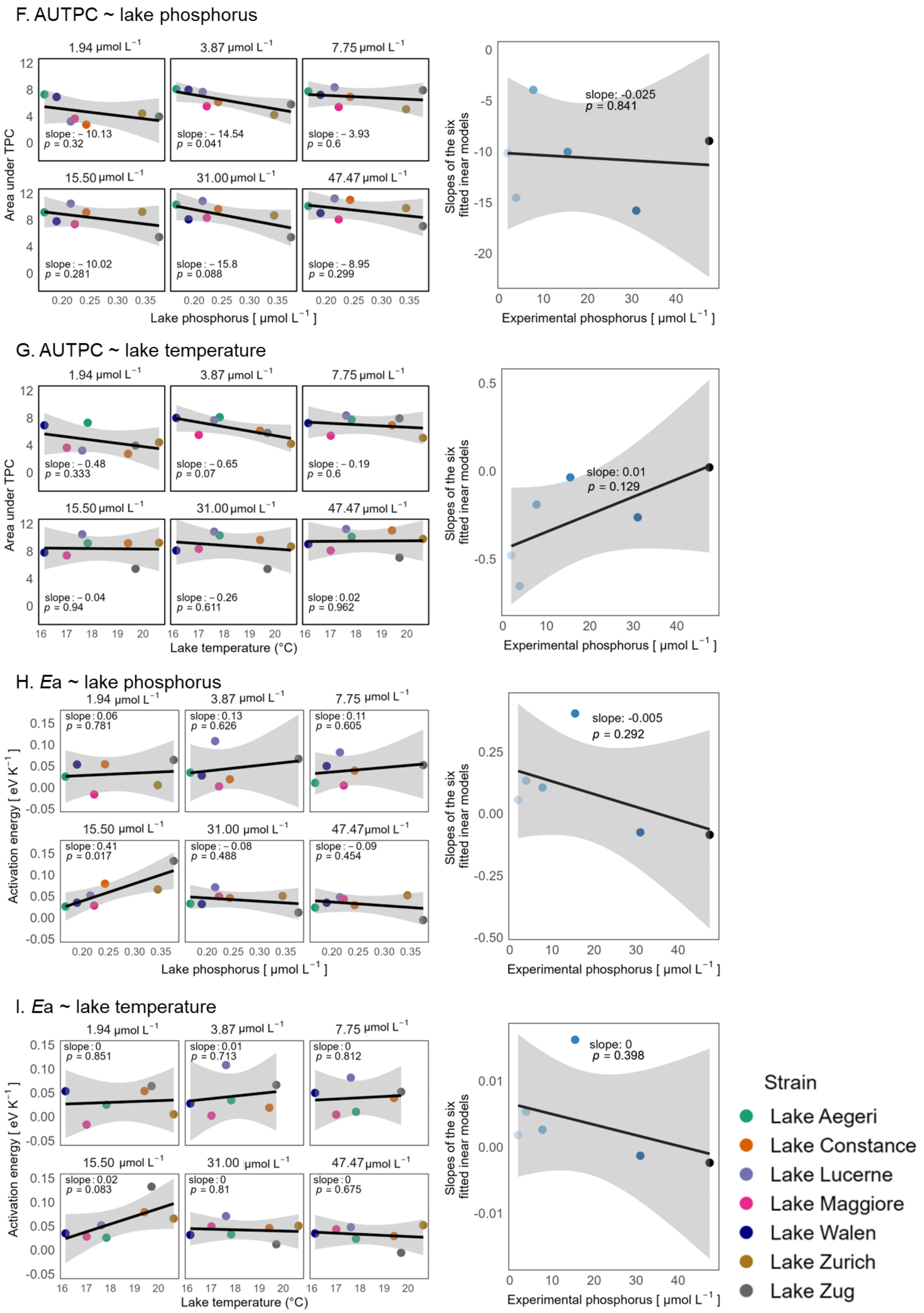

Figure S7. Relationships between thermal traits (A-E) or resource-use traits (F-I) and temperature or phosphorus of lakes where the corresponding strains were from, and how those relationships change with experimental temperature (for resource-use traits) or phosphorus (for thermal traits). The relationship between *P** and lake phosphorus, and how it changes with experimental temperature are presented in Fig. 6.

Table S1. Genetic markers and primers used to assess genetic variability between the eight strains.

Marker	Identifier	Primer	Source
18S	18S_Fwd	CGGTAATTCCAGCTCYV	(Hugerth et al. 2014)
18S	18S_Rev	CCGTCAATTHCTTYAART	(Hugerth et al. 2014)
rbcL	DPrbcL1_F	AAGGAGAAATHAATGTCT	(Jones et al. 2005)
rbcL	DPrbcL7_R	AARCAACCTTGTGTAAGTCTC	(Jones et al. 2005)
rbcL	NDrbcL5_F	CTCAACCATTYATGCG	(Daugbjerg & Andersen 1997)
18S	18S_1132F	AYTTRAAGDAATTGACGG	This study

Table S2. Long PCRSeq strain-specific primers created for further investigation of strain differentiation based on ITS region.

Primer pair	Forward primer name	Forward primer sequence	Reverse primer name	Reverse primer sequence	Band size [bp]	Target strains
1	FraLug15F	tACTGATGTCCTTTCTGGT AAAATTGcGGc	ITS055*RP*	CTC CTT GGT CCG TGT TTC AAG ACG GG	~825~800	Lucerne
2	FraMag5_F2	GGTGGGTTGACATGATGA YcTGWc	ITS055*RP*	CTC CTT GGT CCG TGT TTC AAG ACG GG	~635-645~650	Zurich and Maggiore
3	FraMag5_MAMA_F	TGT CTG TCC TCG TKG AMC TTG GTC ATT	Fra5.8Smid_R	GCA TTT CGC TGC GTT CTT CAT CGT TG	~475~450	Zug and Maggiore
4	Dia_ITS1_2_F1	ATC AYY TAG AGG AAG GWG AAG	FraSeg3_R	TGCAGACACGACATCACGA GAAATCT	~670~720	Seealp
5	Dia_ITS1_2_F2	ATC AYY TAG AGG AAG GWG AAG	FraWag1Agg2_R	GCAACCACGAGCCGaAAc	~245~250	Walen and Aegeri
6	FraCo2G2_F	GGAACTAACATCACAAAC GACCACACA	FraCo2G2_R	GATACTRCGAYTACTACTDT GAGYAGAAAAG	~845~850	Constance

Table S3. NCBI BLAST search of 18S region for species identification. The 18S amplicon sequences matched database sequences for *Fragilaria crotonensis* in the search. The strains from lakes Constance, Maggiore, Zurich, Lucerne and Zug, were consistently identified as the top match. However, in samples from lakes Seealp, Aegeri and Walen, the two genetic markers did not consistently resolve species-level differentiation, potentially due to limitations in the 18S rRNA region's resolution for distinguishing closely related species. The Expect Values (E) for all strains are zero and the query coverages are all 100%.

Origin of strains	Description	Scientific Name	Max Score	Total Score	Percent of identity	Accession Length	Accession
Lakes Aegeri and Walen	*Fragilaria capucina var. mesolepta* isolate HYU-D020 small subunit ribosomal RNA gene, partial sequence	*Fragilaria capucina var. mesolepta*	996	996	99.82%	1655	MH997845.1
	Fragilaria capucina strain CCAC 2678 B small subunit ribosomal RNA gene, partial sequence	*Fragilaria capucina*	996	996	99.82%	560	OL304146.1
	Fragilaria delicatissima partial 18S rRNA gene, strain At135.13	*Ulnaria delicatissima*	996	996	99.82%	1812	AM497721.1
	Fragilaria bidens 18S rRNA gene, strain At170.4	*Fragilaria bidens*	996	996	99.82%	1788	AM497732.1
	Fragilaria capucina strain XZ7.2 small subunit ribosomal RNA gene, partial sequence	*Fragilaria capucina*	996	996	99.82%	1641	OM837397.1

	Centronella reicheltii isolate CCAP 1011/1 small subunit ribosomal RNA gene, partial sequence	*Centronella reicheltii*	996	996	99.82%	1659	MG022762.1
	***Fragilaria crotonensis* strain SAG 28.96 small subunit ribosomal RNA gene, partial sequence**	***Fragilaria crotonensis***	**996**	**996**	**99.82%**	**578**	**OL304142.1**
Lakes Constance, Maggiore, Zurich and Zug	***Fragilaria crotonensis* isolate TCC301 18S ribosomal RNA gene, partial sequence**	***Fragilaria crotonensis***	**1002**	**1002**	**100.00%**	**1694**	**KF959654.1**
Lake Seealp	*Fragilaria delicatissima* partial 18S rRNA gene, strain At135.13	*Ulnaria delicatissima*	1000	1000	100.00%	1812	AM497721.1
	Fragilaria bidens 18S rRNA gene, strain At170.4	*Fragilaria bidens*	1000	1000	100.00%	1788	AM497732.1
	Centronella reicheltii isolate CCAP 1011/1 small subunit ribosomal RNA gene, partial sequence	*Centronella reicheltii*	1000	1000	100.00%	1659	MG022762.1
	***Fragilaria crotonensis* strain SAG 28.96 small subunit ribosomal RNA gene, partial sequence**	***Fragilaria crotonensis***	**1000**	**1000**	**100.00%**	**578**	**OL304142.1**

Lake Lucerne	***Fragilaria crotonensis* small subunit ribosomal RNA gene, partial sequence**	***Fragilaria crotonensis***	**985**	**985**	**99.45%**	**1741**	**AF525662.1**

Table S4. Temperature acclimation. “Day” means how many days before the experiment started, e.g. -8 means 8 days before the experiment. The experiment started on day 0. Temperature groups 1 to 6 correspond to the six temperature levels in the experiment, from low to high. “/” indicates that from that day on, light and/or temperature no longer changed and stayed at that level until the end of the experiment.

Day	Temperature group	Temperature (°C)	Light (μmol photons m^{-2} s^{-1})
-9	1	18	40
	2	20	
	3	20	
	4	20	
	5	20	
	6	20	
-8	1	16	40
	2	20	
	3	20	
	4	20	
	5	20	
	6	22	
-7	1	14	60
	2	18	
	3	20	
	4	20	
	5	22	
	6	24	
-6	1	12	60
	2	16	
	3	20	
	4	22	
	5	24	
	6	26	

-5	1~6	/	80
-4	1~6	/	80
-3	1~6	/	110
-2	1~6	/	/

Table S5. Lake long-term monitoring data summary.

Lake	Monitoring agency	Phosphorus 2018-2022 (µmol L^{-1})	Temperature 2018-2022 (°C)	Monitoring frequency	Period
Aegeri	Amt für Umwelt Kanton Zug	0.17	17.84	4 to 8 times/year	2014-2023
Constance	Institut für Seenforschung LUBW	0.24	19.42	monthly	2000-2023
Lucerne	UWE Kanton Luzern	0.22	17.62	6 times/year	1967-2023
Maggiore (Rogora 2024)	International Commission for the Protection of Italian-Swiss Waters (CIPAIS)	0.22	17.02	monthly	2000-2022
Walen	Wasserversorgung Zürich	0.19	16.14	4 times/year	2014-2023
Zurich	Wasserversorgung Zürich	0.34	20.63	monthly	2000-2023
Zug	Amt für Umwelt Kanton Zug	0.37	19.71	monthly	2014-2023

References

Daugbjerg, N. & Andersen, R.A., 1997. A MOLECULAR PHYLOGENY OF THE HETEROKONT ALGAE BASED ON ANALYSES OF CHLOROPLAST-ENCODED *rbc*L SEQUENCE DATA[1]. *Journal of phycology*, 33(6), pp.1031–1041.

Hugerth, L.W. et al., 2014. Systematic design of 18S rRNA gene primers for determining eukaryotic diversity in microbial consortia. *PloS one*, 9(4), p.e95567.

Jones, H.M. et al., 2005. Life history and systematics of Petroneis (Bacillariophyta), with special reference to British waters. *European journal of phycology*, 40(1), pp.61–87.

Rogora, M., 2024. Water chemistry_Lago Maggiore_Italy. Available at: http://dx.doi.org/10.23728/B2SHARE.FE41F237792B48F19D53DB5258C1BE96.
